# Effect of traditional versus video-assisted problem-based learning on pediatric nursing students’ academic self-efficacy and self-directed learning: implications for pediatric patients care

**DOI:** 10.1186/s12909-026-10037-9

**Published:** 2026-08-11

**Authors:** Fatma Alm Eldeen Goda, Amal Gharib Sabaq, Reda Mohamed Abdullah

**Affiliations:** Pediatric Nursing Department, Faculty of Nursing, Benha University, Benha, Egypt

**Keywords:** Pediatric Nursing, Problem-Based Learning (PBL), Academic Self-Efficacy, Self-Directed Learning Readiness

## Abstract

**Background:**

High-quality pediatric care depends on well-prepared nursing students. Innovative educational strategies, such as problem-based learning (PBL), have gained recognition for their potential to enhance students’ self-efficacy and self-directed learning, which are essential for providing safe and effective care to children.

**Objective:**

This study aims to compare the effects of traditional PBL versus video-assisted PBL on pediatric nursing students’ academic self-efficacy and self-directed learning in order to explore their implications for pediatric patient care.

**Methods:**

An experimental research study was conducted at the Faculty of Nursing, Benha University. A systematic random sample of 150 pediatric nursing students was utilized. Participants were evaluated using a structured interviewing questionnaire for knowledge, the Academic Self-Efficacy Scale, and the Self-Directed Learning Readiness Scale. The study compared experimental groups (Traditional PBL and Video-assisted PBL) against a control group.

**Results:**

Following the intervention, significant improvements were observed in total knowledge, academic self-efficacy, and self-directed learning readiness among both experimental groups compared with the control group (*P* < 0.001). The video-assisted PBL group achieved the highest post-intervention mean scores in total knowledge (77.30 ± 4.42), academic self-efficacy (72.16 ± 6.18), and self-directed learning readiness (118.38 ± 3.76), followed by the traditional PBL group (72.22 ± 6.96, 67.70 ± 7.78, and 113.84 ± 11.72, respectively), whereas the control group demonstrated the lowest scores (59.98 ± 12.89, 45.74 ± 5.88, and 84.86 ± 10.28, respectively). Post hoc analyses further confirmed that video-assisted PBL significantly outperformed traditional PBL and conventional teaching across all measured outcomes (*P* ≤ 0.001).

**Conclusion:**

This study provides evidence that both traditional and video-assisted PBL are effective in improving students’ learning outcomes. However, Video-assisted PBL is superior in enhancing knowledge, academic self-efficacy, and self-directed learning readiness, offering a more effective strategy for nursing education.

**Trial registration:**

Not applicable.

**Supplementary Information:**

The online version contains supplementary material available at 10.1186/s12909-026-10037-9.

## Highlights

⚉ Video-assisted and traditional PBL improve pediatric nursing students’ knowledge and problem-based learning skills.

⚉ Video-assisted PBL significantly enhances self-directed learning readiness and academic competence.

⚉ Video-assisted PBL demonstrates superior educational outcomes compared with traditional PBL and conventional teaching.

⚉ Technology-supported PBL may improve the quality of pediatric nursing education and patient care

**Supplementary Information:**

The online version contains supplementary material available at 10.1186/s12909-026-10037-9.

## Introduction

The landscape of nursing education is currently undergoing a transformative paradigm shift, transitioning from conventional instructional methods toward dynamic, clinically integrated models. This evolution aims to prepare nursing professionals capable of navigating the intricate demands of modern healthcare with high precision. However, traditional lecture-based approaches often struggle to bridge the gap between theoretical knowledge and clinical practice, particularly in specialized fields like pediatrics, where adaptability and critical thinking are paramount [1, 2]. Given the rapid expansion of medical evidence and the integration of sophisticated technology, there is a global push toward learner-centered methodologies that foster active engagement and ensure students are ready for complex, real-world clinical scenarios [3, 4].

Problem-Based Learning (PBL) has emerged as a cornerstone of this educational reform. Rooted in constructivist principles, PBL shifts the focus from rote memorization to analytical inquiry and proactive engagement [5, 6]. In this framework, students take responsibility for their own learning by defining objectives and collaborating in small groups to resolve clinical dilemmas, while educators transition from primary lecturers to guided facilitators [7, 8]. This role is crucial, as nurse educators must design curricula that resonate with contemporary healthcare trends and equip graduates with the competencies needed to shape the future of nursing [9–11]. Beyond instruction, facilitators in a PBL environment provide essential mentorship and constructive feedback to cultivate effective teamwork [12, 13].

Central to this pedagogical shift is the development of Self-Directed Learning (SDL) and Academic Self-Efficacy. SDL represents the learner’s autonomous capacity to identify educational needs, set precise goals, and evaluate personal progress—a necessity in today’s complex healthcare systems [14, 15]. Recent evidence suggests that PBL, particularly when augmented by video-assisted technology, acts as a vital catalyst for SDL by enhancing student agency and intrinsic motivation [16, 17]. Furthermore, academic self-efficacy, the confidence students have in their ability to execute complex tasks, is reinforced through the mastery experiences and peer synergy provided by innovative PBL frameworks [18, 19]. High levels of self-efficacy are strongly correlated with superior clinical performance and professional readiness [20, 21].

The necessity of these competencies is most evident in pediatric nursing, where practitioners must synthesize specialized knowledge with advanced clinical reasoning to care for vulnerable populations [22, 23]. Ensuring patient safety and maintaining effective therapeutic communication with families requires precision and the ability to respond to rapid clinical changes [24, 25]. Consequently, educational strategies that bolster SDL and self-efficacy are instrumental in refining students' decision-making proficiency, ultimately leading to enhanced pediatric patient outcomes [26].

Despite the documented advantages of PBL in promoting critical thinking and teamwork [27, 28], there remains a significant research gap regarding its application in pediatric nursing education within the Egyptian context, specifically in Benha City. Most existing literature focuses on traditional text-based PBL, with limited exploration of video-assisted approaches. This study addresses this gap by comparing the effectiveness of text-based and video-assisted PBL on the academic self-efficacy and self-directed learning of pediatric nursing students, thereby contributing to the improvement of clinical preparedness and the quality of care provided to children.

### Aim of the study

This study aimed to compare the effects of traditional PBL versus video-assisted PBL on pediatric nursing students’ academic self-efficacy and self-directed learning, and to explore the implications for pediatric patient care outcomes.

### Hypotheses

- H1: Experimental groups who will receive traditional and video-assisted problem-based learning are expected to have higher knowledge mean score than the control group.
- H2: Experimental groups who will receive traditional and video-assisted problem-based learning are expected to have higher academic self-efficacy mean score than the control group.
- H3: Experimental groups who will receive traditional and video-assisted problem-based learning are expected to have higher self-directed learning mean score than the control group.

## Materials and methods

### Research design

A randomized controlled experimental design was employed to evaluate the causal relationships between the study variables. This structured approach provided a comprehensive framework for data collection and analysis over a six-month period, from October 2024 to March 2025. To ensure methodological rigor and minimize bias, the study followed the CONSORT guidelines for reporting randomized trials [29].

### Setting

The study was conducted in the pediatric nursing classroom at the Faculty of Nursing, Benha University. The facility was specifically equipped with the necessary instructional technology and collaborative spaces to support the effective implementation of both traditional and video-assisted problem-based learning (PBL) sessions.

### Sampling and participants

The total population consisted of 544 third-year pediatric nursing students. The sample size was calculated using Slovin’s formula.

Formula:

$$n=\frac{N}{1+Ne2}$$

**where:**

- **N** = Total population size = 171
- **e** = Margin of error = 0.05 (at 95% confidence level)
- **n** = Resulting sample size

Substituting into the formula: n = 544/(1 + 544 × 0.05^2) = 231.

**Figure. 1.**
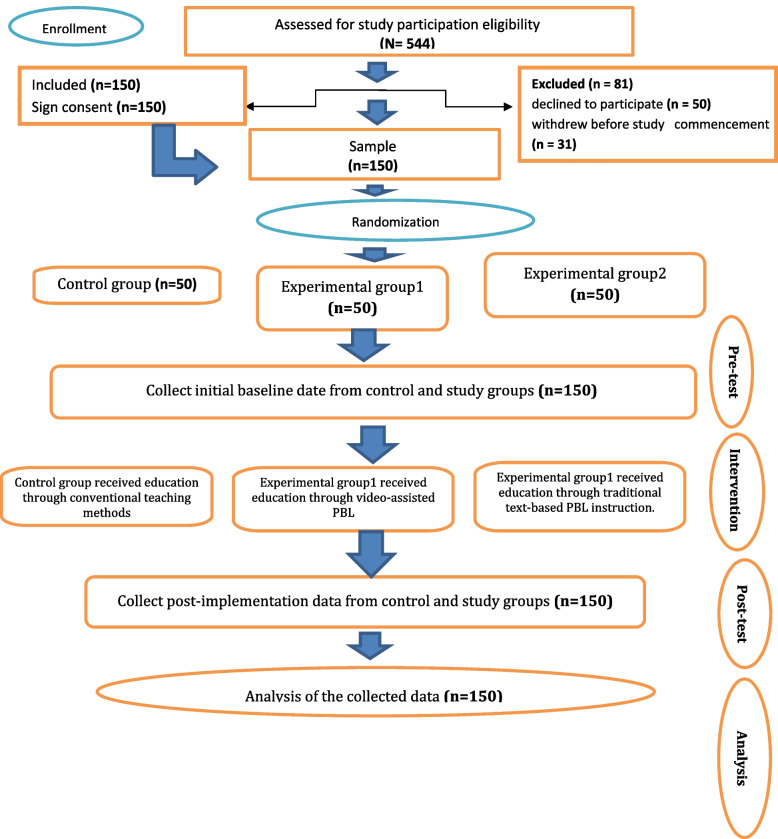
Flow of the study procedure

A systematic random sampling technique was used to select the required sample. The sampling interval was calculated as approximately 2 (544/231 = 2.35). Therefore, every second student was selected from the population register following a randomly chosen starting point. In the present study, the random starting point was the third student on the register. Out of the 231 students assessed for eligibility, 150 provided informed consent, while 50 declined and 31 withdrew due to personal commitments. Thus, 150 participants were included in the final analysis (Fig. 1).

A post hoc power analysis was performed using G*Power software version 3.1.9.7 to evaluate whether the final sample size was sufficient to detect the observed differences among the three study groups. The analysis was based on a one-way fixed-effects ANOVA with three groups, a total sample size of 150 participants (50 participants per group), an alpha level of 0.05, and the observed effect size (f = 0.832) derived from the total knowledge score ANOVA results. The achieved power was > 0.99, indicating adequate statistical power to detect the observed group differences.

### Randomization and blinding

Participants (*n* = 150) were randomly assigned by an independent researcher to ensure impartiality. The Control Group (*n* = 50) received conventional teaching, while the Experimental Group (*n* = 100) was subdivided into two equal cohorts: Video-assisted PBL (*n* = 50) and Traditional Text-based PBL (*n* = 50). This allocation ensured an equal distribution across all three study arms, enhancing statistical power for robust comparison. To ensure objective assessment, outcome evaluators were blinded to the group assignments. A baseline analysis confirmed no significant differences between the three groups regarding age or academic background (*P* > 0.05), ensuring initial comparability.

#### Data collection tools

Data were collected using three tools:

##### Tool (I): A structured interviewing questionnaire

It was designed by the researchers after reviewing recent and relevant literature and evidence-based guidelines related to problem-based learning methodology and pediatric nursing care [30–47]. It was written in English language and composed of the following parts:

##### Part (I): Characteristics of the studied students

It was concerned with characteristics of the studied pediatric nursing students including age, gender, place of residence, previous qualification and attending previous training programs by using the PBL (5 items).

##### Part (II): Students' knowledge assessment questionnaire (pre/posttest)

The structured interviewing questionnaire was developed by the researchers based on an extensive review of relevant national and international literature and evidence-based nursing guidelines [30–47]. The questionnaire is provided as a supplementary file.

It comprised 100 closed-ended multiple-choice questions divided into two domains:

• Knowledge related to the PBL methodology (20 items): covering the definition, design, goals, student and teacher roles, PBL steps, scenario criteria, common difficulties, and advantages/disadvantages based on previous literature [30–33].
• Pediatric nursing knowledge (80 items): covering diabetes mellitus, bronchial asthma, acute rheumatic fever, beta thalassemia, and kwashiorkor, developed according to relevant evidence-based guidelines and literature [34–47].

Correct answers were scored as “1” and incorrect or “don’t know” responses as “0”.

For the purpose of evaluating learning outcomes among the study groups, only the pediatric nursing knowledge domain (80 items) was used in the comparative analyses, whereas the PBL knowledge items were analyzed separately because they assessed knowledge of the instructional method rather than subject-matter knowledge.

Knowledge levels were categorized as poor (< 60%), average (60– < 80%), and good (≥ 80%). The tool demonstrated acceptable reliability (Cronbach’s α = 0.784).

##### Tool II: Academic Self-Efficacy Scale (Pre/Post-Test)

Academic self-efficacy of pediatric nursing students in both experimental and control groups was assessed using the scale adopted from Kim and Park [48]. The scale included three domains: task difficulty preference (8 items), self-confidence (8 items), and self-regulatory efficacy (9 items), rated on a 3-point Likert scale (always = 3, sometimes = 2, never = 1), with total scores ranging from 25 to75. Higher scores indicated greater academic self-efficacy and were categorized as low (<45), moderate (45–<56), and high (≥56). The scale demonstrated good reliability with a Cronbach’s α of 0.875.

##### Tool III: SDL Readiness Scale (Pre/Post-Test)

Readiness for self-directed learning among pediatric nursing students was assessed using the 40-item Self-Directed Learning Readiness Scale (SDLRS) developed by Fisher et al. [49]. The scale was adopted in its original form without any modifications and comprised three subscales: self-management (13 items), desire for learning (12 items), and self-control (15 items). Items were rated on a 3-point Likert scale (always = 3, sometimes = 2, and never = 1), with total scores ranging from 40 to 120. Scores were categorized as low (< 72), moderate (72– < 90), and high (≥ 90). The reliability of the scale was re-assessed in the current study and demonstrated excellent internal consistency (Cronbach’s α = 0.925).

### Pilot study

A pilot study was conducted on 10% of the total sample (*n* = 15 students) to evaluate the clarity, relevance, feasibility, and clinical applicability of the research tools. This phase ensured the logical sequence of questions and the consistency of the data collection process. Additionally, it provided an accurate estimate of the time required to complete the three assessment tools, which was approximately 45–60 min per student, considering the number of items and the time needed for students to carefully read and respond to each question. Based on the pilot analysis, no modifications were necessary; therefore, the tools were finalized in their original form. The participants involved in the pilot study were included in the main study sample as no changes were made to the instruments. This phase was carried out throughout October 2024.

### Content validity

Tools of data collection were designed and submitted to a jury of the experts (3 Professors) in the field of Pediatric Nursing Specialty from the Faculty of Nursing/Benha University to test the content validity of tools and judge its clarity, comprehensiveness, relevance, simplicity and accuracy. Based on experts' comments and recommendations, minor modifications had been made such as rephrasing, rearrangement or deleting some sentences to reach the final version of the tools. The tools were considered as valid from the experts' perspective.

### Field work

The study adopted a three-tiered analytical approach to fulfill its aims, categorized into: (I) the preliminary (pre-application) phase, (II) the active implementation phase, and (III) the evaluative (post-application) phase.

### Pre-application phase

Prior to implementation, the study protocol, educational strategy, structured clinical scenarios, and practical implementation plan were reviewed and approved by the Head of the Pediatric Nursing Department, Faculty of Nursing, Benha University. The intervention was conducted within the framework of the existing pediatric nursing curriculum without modifying the official course objectives or content.

Prior to the intervention, the researchers attended the study setting and met the participants according to their academic timetable, three days per week from 9:00 a.m. to 3:00 p.m. to inform them about the study aim, procedures, and tools before their enrollment. Participation began upon submission of signed written informed consent. Subsequently, all participants were interviewed to complete the questionnaire and collect pretest baseline data using Tools (I, II, & III). These interviews conducted individually each interview required approximately 10–15 min for students to complete all three tools. The baseline data collection process took approximately one month, from the beginning to the end of October 2024.

Following the acquisition of baseline data, the researchers developed the educational content related to selected pediatric conditions, including diabetes mellitus, bronchial asthma, acute rheumatic fever, beta thalassemia, and kwashiorkor. The educational content and clinical scenarios were developed based on the existing pediatric nursing curriculum and supported by evidence-based guidelines and authoritative pediatric references, including the American Academy of Pediatrics guidelines, American Diabetes Association Standards of Care, Global Initiative for Asthma recommendations, and pediatric nursing textbooks. The identified learning needs, knowledge gaps, and deficiencies revealed by the baseline assessment were translated into the aims and learning objectives of the educational content. The educational content was identical across the intervention groups and was delivered through conventional teaching methods for the control group, whereas the experimental groups received the same content using either video-assisted PBL or traditional text-based PBL. The researchers carefully prepared and arranged the learning environment to support the delivery of PBL sessions. All necessary resources were provided to ensure an effective learning experience, including chairs arranged to facilitate face-to-face group discussions, writing materials, video display devices, appropriate lighting control, and audiovisual facilities to enhance learning effectiveness.

### Control group (*n* = 50)

The educational content was delivered through face-to-face lectures combined with group discussions and covered five pediatric theoretical lectures named (diabetes mellitus, bronchial asthma, acute rheumatic fever, beta thalassemia, & kwashiorkor). The sessions were conducted according to the official academic schedule, delivered by pediatric nursing staff, and held once weekly on Mondays, with each lecture lasting two hours. The researchers attended all lectures as observers to monitor implementation process and ensure students’ adherence. The intervention lasted approximately five weeks, from early November 2024 to mid-December 2024.

### Active implementation phase

#### Workshop and orientation

Prior to the intervention, a one-day orientation workshop (2 h) was conducted for all students in the experimental groups (*n* = 100). The workshop aimed to familiarize participants with the principles of Problem-Based Learning (PBL), their evolving roles as active learners, and the specific dynamics of the upcoming sessions.

#### Group allocation

Following the workshop, the 100 participants were randomly assigned into two equal cohorts:

- Experimental Group I (Video-assisted PBL, n = 50): Educated using AI-generated clinical video scenarios.
- Experimental Group II (Traditional Text-based PBL, n = 50): Educated using standardized written case scenarios. Each group was further subdivided into five small teams (10 students per team) to facilitate intensive peer discussion and collaborative problem-solving.

### Development of PBL scenarios and AI-Video production

Five realistic pediatric clinical scenarios were meticulously developed based on the learning objectives of the selected topics (Diabetes Mellitus, Bronchial Asthma, Acute Rheumatic Fever, Beta Thalassemia, and Kwashiorkor).

To ensure the highest quality of the intervention, these scenarios underwent a rigorous validation process. They were reviewed by the study supervisors and further evaluated by a panel of three academic professionals, including two pediatric nursing specialists and one pediatric medicine specialist. Additionally, the scenarios were pilot-tested by 15 students to assess usability, visibility, structure, and layout. Based on the experts' feedback and pilot results, minor modifications were applied, including rewording, reorganizing content, and adjusting the format to finalize the scenarios.

For the Text-based Group: The finalized scenarios were presented as structured written narratives.

For the Video-Assisted PBL Group: The researcher converted the developed clinical scenarios into cinematic scripts designed for visual storytelling. These scripts were subsequently produced as high-fidelity educational videos using artificial intelligence–based multimedia generation tools under the supervision of a specialist engineer. Visual scenes, digital avatars, and simulated clinical environments were created using Flow AI, while AI-generated voice narration was produced using ElevenLabs. All videos were reviewed by the research team prior to implementation to ensure consistency with the intended learning objectives, clinical accuracy, and educational quality. The AI-generated videos depicted realistic pediatric clinical cases, including symptom presentation, family interactions, and nursing challenges. This approach was intended to promote student engagement, enhance clinical reasoning, and facilitate a deeper understanding of real-world pediatric nursing situations.

The PBL intervention was implemented through two face-to-face sessions (Saturday and Wednesday) based on Barrows’ PBL Model [50] (Table 1).

**Table 1:** The Barrows PBL Model

Steps	What to do?	Details
Step 1	Introduce the Problem	An ill-structured, realistic problem is presented
Step 2	Brainstorming	Students analyze the problem, discuss initial thoughts, and identify what they already know
Step 3	Define Learning Objectives	Students identify what they need to learn to understand and solve the problem
Step 4	Self-Study	Individual research is conducted on the identified learning gaps
Step 5	Re-evaluating & Applying	The group meets to share new knowledge, apply it to the problem, and refine their solutions
Step 6	Reflection	Students evaluate their problem-solving process and the new knowledge acquired

Each session lasted approximately 1.5–2 h (4 h in total). During the first session (on Saturday), Students were presented with a pediatric clinical scenario (problem case) designed to stimulate discussion, critical thinking, and analysis. They were encouraged to analyze the problem, discuss their initial thoughts, identify what they already knew, and determine what they needed to learn to better understand and solve the problem. At the end of the session, students were reminded of the importance of adhering to the scheduled sessions. During the second session on Wednesday, the students conducted individual research to identify learning gaps, begin sharing new knowledge, and apply it to solve the problem. At the end of the session, students’ reflections were collected to evaluate their problem-solving process and the acquisition of new knowledge. It should be noted that the researchers were present as facilitators during both sessions. The session steps were repeated over five weeks, with one case scenario addressed each week, covering the five predetermined pediatric topics for all experimental groups.

### Evaluative phase (Post-Application)

Immediately following the five-week intervention, the effectiveness of the teaching methods was assessed using the three study tools (Tools I, II, and III). This post-test evaluation aimed to measure significant changes in students' knowledge, academic self-efficacy, and self-directed learning readiness compared to their baseline scores.

### Ethical considerations

The study was conducted following approval from the Scientific Research Ethical Committee at the Faculty of Nursing, Benha University (Ethics code: REC-PN.P77) prior to data collection. Written informed consent was obtained from all participating students after providing a clear explanation of the study’s purpose, procedures, and expected outcomes. Participation was voluntary, and students were allowed to withdraw at any time without any consequences. Confidentiality and anonymity were maintained through data coding, and all collected information was used exclusively for research purposes. The study adhered to ethical principles by respecting participants’ values, cultural backgrounds, and personal beliefs. At the conclusion of the study, the educational materials and PBL videos were made accessible to students in the other groups.

### Statistical analysis

The collected data were revised, organized, coded, tabulated, and analyzed using the Statistical Package for Social Sciences (SPSS) version 25. Quantitative data were expressed as mean and standard deviation (SD), while qualitative data were presented as frequencies and percentages. Qualitative variables were analyzed using the Chi-square test (χ^2^) and Fisher’s Exact Test (FET). Pearson’s correlation coefficient (r) was used to determine the relationships between study variables. Comparisons of mean scores between the three study groups were performed using one-way analysis of variance (ANOVA). When statistically significant differences were identified, post hoc multiple comparison tests were conducted to determine differences between individual groups. The observed differences were considered non-significant at *P* > 0.05, significant at *P* < 0.05, and highly significant at *P* < 0.001.

## Results

Table 2: Illustrates the baseline characteristics of the participants across the three groups. No statistically significant differences were observed among the video-assisted PBL, traditional PBL, and control groups regarding age, gender, residence, and previous qualifications (*p* > 0.05), indicating that the groups were comparable before intervention.

**Table 2:** Distribution of the studied students according to their socio-demographic characteristics among the video-assisted PBL, traditional PBL, and control groups (*n* = 150)

**Variables**/**Groups**	**Study groups**				**Control group (*n* = 50)**		**Test of significance**	***P*-value**
	**Video-assisted PBL group (*n* = 50)**		**Traditional PBL group (*n* = 50)**					
	**No**	**%**	**No**	**%**	**No**	**%**		
**Age/years**								
- < 20	2	4.0	1	2.0	4	8.0	χ^2^ = 3.05	0.550
- 20- < 22	42	**84.0**	44	**88.0**	43	**86.0**		
- 22- < 24	6	12.0	5	10.0	3	6.0		
**Mean ± SD**	**20.72 ± 0.88**		**20.76 ± 0.69**		**20.52 ± 0.89**		F = 1.22	0.298
**Gender**								
- Male	21	42,0	16	32.0	14	28	χ^2^ = 2.32	0.314
- Female	29	**58.0**	34	**68.0**	36	**72.0**		
**Place of residence**								
- Urban	9	18.0	10	20.0	7	14.0	χ^2^ = 0.65	0.722
- Rural	41	**82.0**	40	**80.0**	43	**86.0**		
**Qualification before attachment to the faculty**								
- Secondary school	33	**66.0**	30	**60.0**	32	**64.0**	χ^2^ = 0.40	0.818
- Associate degree of nursing	17	34.0	20	40.0	18	36.0		
**Attending previous training program about problem based learning**								
- Yes	0	0.0%	0	0.0%	0	0.0%	Not applicable*	
- No	50	**100.0**	50	**100.0**	50	**100.0**		

χ^2^ = Chi-square test; F = One-way ANOVA test

*Not applicable because all students reported no previous training about problem-based learning

Table 3: demonstrates the comparison of students’ subtotal knowledge scores regarding diabetes, asthma, rheumatic fever, thalassemia, and kwashiorkor before and after the intervention. Before implementation, no statistically significant differences were observed among the three groups across all pediatric topics (*p* > 0.05), indicating baseline comparability of the study groups. After implementation, statistically significant differences were detected among the three groups in all knowledge domains (*p* < 0.001). Students in the video-assisted PBL group achieved the highest mean knowledge scores across all studied pediatric topics, followed by the traditional PBL group, while the control group showed the lowest scores. These findings indicate the effectiveness of PBL approaches, particularly video-assisted PBL, in improving pediatric nursing students’ knowledge.

**Table 3:** Mean scores of students’ knowledge regarding pediatric nursing topics among the video-assisted PBL, traditional PBL, and control groups before and after implementation (*n* = 150)

Items	Maximum score	Time point	Study groups		Control group (*n* = 50)	F	*P*-value
			**Video-assisted PBL group** **(*n* = 50)**	**Traditional PBL group** **(*n* = 50)**			
			**Mean ± SD**	**Mean ± SD**	**Mean ± SD**		
**Diabetes**	**20**	Before implementation	7.60 ± 4.04	6.70 ± 3.86	6.86 ± 4.28	0.70	0.499
		After implementation	18.34 ± 2.45	18.32 ± 2.19	14.24 ± 4.25	28.96	< 0.001
**Asthma**	**20**	Before implementation	7.34 ± 4.43	8.16 ± 3.80	7.00 ± 4.34	1.01	0.368
		After implementation	19.66 ± 1.12	17.76 ± 2.88	14.68 ± 5.20	25.89	< 0.001
**Rheumatic fever**	**15**	Before implementation	5.14 ± 3.98	4.78 ± 3.82	5.06 ± 3.81	0.12	0.888
		After implementation	14.58 ± 1.36	13.52 ± 1.92	11.78 ± 3.50	16.90	< 0.001
**Thalassemia**	**15**	Before implementation	4.94 ± 2.97	4.44 ± 3.92	4.70 ± 3.21	0.27	0.762
		After implementation	14.84 ± 0.47	13.48 ± 1.78	11.48 ± 2.53	43.77	< 0.001
**Kwashiorkor**	**10**	Before implementation	3.54 ± 2.57	3.46 ± 2.65	3.10 ± 2.82	0.38	0.683
		After implementation	9.88 ± 0.39	9.14 ± 1.21	7.80 ± 2.46	21.77	< 0.001

F = One-way ANOVA test

Statistically significant difference: *p* < 0.001

No statistically significant difference: *p* > 0.05

Table 4: demonstrates the mean total knowledge scores of students in the study and control groups before and after the implementation of the educational interventions. Before implementation, no statistically significant difference was observed among the three groups regarding total knowledge scores (F = 0.183, *P* = 0.833), indicating baseline homogeneity of the groups. After implementation, a highly statistically significant difference was found among the groups (F = 50.795, *P* < 0.001), with students in the video-assisted PBL group achieving the highest mean knowledge scores, followed by the traditional PBL group, while the control group showed the lowest improvement. These findings indicate the effectiveness of PBL approaches, particularly video-assisted PBL, in enhancing pediatric nursing students’ knowledge.

**Table 4:** Mean total knowledge scores among the video-assisted PBL, traditional PBL, and control groups before and after implementation (*n* = 150)

Items	Total score	Time point	Video-assisted PBL group (*n* = 50)	Traditional PBL group (*n* = 50)	Control group (*n* = 50)	F	*P*-value
			**Mean ± SD**	**Mean ± SD**	**Mean ± SD**		
**Total knowledge**	**80**	Before implementation	28.56 ± 15.63	27.54 ± 14.49	26.72 ± 15.59	0.18	0.833
		After implementation	77.30 ± 4.42	72.22 ± 6.96	59.98 ± 12.89	50.80	< 0.001

F = One-way ANOVA test

Statistically significant difference: *p* < 0.001

No statistically significant difference: *p* > 0.05

Table 5: presents the results of the post hoc LSD multiple comparisons of students’ mean knowledge scores among the three groups before and after the implementation of the educational intervention. Before implementation, no statistically significant differences were detected among the Video-assisted PBL, Traditional PBL, and control groups (*P* > 0.05), indicating baseline comparability of the groups regarding students’ knowledge levels. After implementation, significant differences were observed among all pairwise comparisons (*P* ≤ 0.001). The Video-assisted PBL group demonstrated significantly higher mean knowledge scores compared with both the Traditional PBL group (mean difference = 8.10) and the control group (mean difference = 23.14). Additionally, the Traditional PBL group showed significantly higher knowledge scores than the control group (mean difference = 15.04). These findings indicate that both PBL approaches improved students’ knowledge; however, the Video-assisted PBL approach achieved the greatest improvement compared with traditional PBL and conventional teaching.

**Table 5:** LSD post hoc multiple comparisons of students’ knowledge scores among the video-assisted PBL, traditional PBL, and control groups before and after implementation (*n* = 150)

Time point	(I) group	(J) group	Mean Difference (I-J)	*P*-value
**before implementation**	Video-assisted PBL group	Traditional PBL group	1.94	0.568
	Video-assisted PBL group	Control group	2.46	0.469
	Traditional PBL group	Video-assisted PBL group	−1.94	0.568
	Traditional PBL group	Control group	0.52	0.878
	Control group	Video-assisted PBL group	−2.46	0.469
	Control group	Traditional PBL group	−0.52	0.878
**after implementation**	Video-assisted PBL group	Traditional PBL group	8.10	< 0.001
	Video-assisted PBL group	Control group	23.14	< 0.001
	Traditional PBL group	Video-assisted PBL group	−8.10	< 0.001
	Traditional PBL group	Control group	15.04	< 0.001
	Control group	Video-assisted PBL group	−23.14	< 0.001
	Control group	Traditional PBL group	−15.04	< 0.001

*LSD* Least Significant Difference post hoc test

No statistically significant difference: *p* > 0.05

Statistically significant difference: *p* < 0.001

**Fig. 2:**
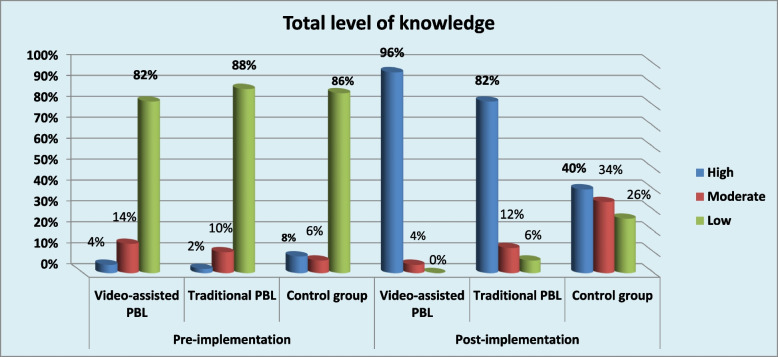
Total nursing students' knowledge level among among the video-assisted PBL, traditional PBL, and control groups (*n* = 150)

Figure 2: illustrates the distribution of students’ total knowledge levels among the Video-assisted PBL, Traditional PBL, and control groups before and after implementation. Before the intervention, the majority of students in all groups had low knowledge levels, with no students achieving a high knowledge level except a small proportion in both PBL groups, indicating comparable baseline knowledge levels. After implementation, a marked improvement in knowledge levels was observed among students in both intervention groups. The Video-assisted PBL group showed the greatest improvement, with the majority of students achieving a high knowledge level, followed by the Traditional PBL group. In contrast, although the control group demonstrated some improvement, a considerable proportion of students remained at moderate and low knowledge levels. These findings highlight the superior improvement in students’ knowledge levels following the Video-assisted PBL approach compared with Traditional PBL and conventional teaching.

Table 6: presents the mean subtotal and total scores of students’ academic self-efficacy among the Video-assisted PBL, Traditional PBL, and control groups before and after implementation. Before the intervention, no statistically significant differences were found among the three groups regarding task-difficulty preference, self-confidence, self-regulatory efficacy, or total academic self-efficacy scores (*P* > 0.05), indicating baseline equivalence among the groups.

**Table 6:** Mean subtotal and total scores of students' academic self-efficacy in both among the video-assisted PBL, traditional PBL, and control groups pre and post implementation (*n* = 150)

Items	Maximum score	**Time point**	Study groups		Control group (*n* = 50)	F	*P*-value
			Video-assisted PBL group (*n* = 50)	Traditional PBL group (*n* = 50)			
			Mean ± SD	Mean ± SD	Mean ± SD		
Task-difficulty preference	24	Before implementation	14.36 ± 2.92	13.58 ± 2.37	14.02 ± 2.34	1.17	0.313
		After implementation	23.26 ± 2.14	21.38 ± 2.59	14.98 ± 2.58	157.66	< 0.001
Self-confidence	24	Before implementation	12.98 ± 3.50	12.44 ± 3.14	12.42 ± 3.02	0.49	0.616
		After implementation	23.04 ± 2.29	21.82 ± 3.04	13.70 ± 3.13	159.50	< 0.001
Self-regulatory efficacy	27	Before implementation	16.88 ± 3.40	16.70 ± 3.34	16.14 ± 3.08	0.69	0.501
		After implementation	25.86 ± 2.05	24.50 ± 3.07	17.06 ± 2.28	178.63	< 0.001
Total academic self-efficacy	75	Before implementation	44.22 ± 6.74	42.72 ± 5.54	42.58 ± 4.98	1.23	0.296
		After implementation	72.16 ± 6.18	67.70 ± 7.78	45.74 ± 5.88	225.25	< 0.001

F = One-way ANOVA test

No statistically significant difference: *p* > 0.05

Statistically significant difference: *p* < 0.001

After implementation, statistically significant differences were observed among the three groups in all dimensions of academic self-efficacy and in the total score (*P* < 0.001). The Video-assisted PBL group achieved the highest mean scores across all academic self-efficacy domains, followed by the Traditional PBL group, whereas the control group showed the lowest improvement. The total academic self-efficacy score increased markedly in the Video-assisted PBL group compared with the Traditional PBL and control groups. These findings indicate that both PBL approaches enhanced students’ academic self-efficacy; however, the Video-assisted PBL approach demonstrated a greater positive impact on improving students’ confidence in managing learning tasks, self-regulation, and overall academic self-efficacy.

Table 7: Post hoc analysis revealed no significant differences in academic self-efficacy scores among groups at baseline (*p* > 0.05). Following implementation, significant differences were found between all groups, with the highest scores observed among students who received video-assisted PBL, followed by traditional PBL and control groups (*p* ≤ 0.001). This suggests the superior effectiveness of video-assisted PBL in enhancing students’ academic self-efficacy.

**Table 7:** LSD Post hoc multiple comparisons of students’ academic self-efficacy scores among the video-assisted PBL, traditional PBL, and control groups pre and post implementation (*n* = 150)

Time point	(I) group	(J) group	Mean Difference (I-J)	*P*-value
**before implementation**	Video-assisted PBL group	Traditional PBL group	1.50	0.198
	Video-assisted PBL group	Control group	1.64	0.160
	Traditional PBL group	Video-assisted PBL group	−1.50	0.198
	Traditional PBL group	control group	0.14	0.904
	Control group	Video-assisted PBL group	−1.64	0.160
	Control group	Traditional PBL group	−0.14	0.904
**after implementation**	Video-assisted PBL group	Traditional PBL group	4.46	0.001**
	Video-assisted PBL group	Control group	26.42	< 0.001**
	Traditional PBL group	Video-assisted PBL group	−4.46	0.001**
	Traditional PBL group	Control group	21.96	< 0.001**
	Control group	Video-assisted PBL group	−26.42	< 0.001**
	Control group	Traditional PBL group	−21.96	< 0.001**

*LSD* Least Significant Difference post hoc test

No statistically significant difference: *p* > 0.05

Statistically significant difference: *p* ≤ 0.05

Highly statistically significant difference: *p* < 0.001

Table 8: illustrates the mean subtotal and total scores of students’ self-directed learning readiness among the video-assisted PBL, traditional PBL, and control groups before and after implementation. Before implementation, no statistically significant differences were observed among the three groups regarding self-management, desire for learning, self-control, or total self-directed learning readiness scores (*p* > 0.05), indicating baseline homogeneity among the studied groups.

**Table 8:** Mean subtotal and total scores of students’ self-directed learning readiness among the video-assisted PBL, traditional PBL, and control groups before and after implementation (*n* = 150)

Items	Maximum score	Time point	Study groups		Control group (n = 50)	F	P-value
			Video-assisted PBL group (n = 50)	Traditional PBL group (n = 50)			
			Mean ± SD	Mean ± SD	Mean ± SD		
**Self-management**	39	Before implementation	25.47 ± 2.60	25.06 ± 1.30	25.86 ± 1.60	2.19	0.116
		After implementation	38.42 ± 1.50	37.02 ± 3.91	27.92 ± 4.22	137.86	< 0.001
**Desire for learning**	36	Before implementation	24.60 ± 2.12	24.08 ± 2.25	24.18 ± 1.75	0.91	0.406
		After implementation	35.30 ± 1.62	34.00 ± 4.00	25.02 ± 3.53	151.37	< 0.001
**Self-control**	45	Before implementation	29.32 ± 2.57	29.84 ± 1.61	29.98 ± 2.52	1.17	0.314
		After implementation	44.66 ± 1.39	42.82 ± 4.73	31.92 ± 5.20	138.39	< 0.001
**Total self-directed learning readiness**	120	Before implementation	79.34 ± 5.29	78.98 ± 3.94	80.02 ± 4.29	0.68	0.510
		After implementation	118.38 ± 3.76	113.84 ± 11.72	84.86 ± 10.28	192.85	< 0.001

F = One-way ANOVA test

No statistically significant difference: *p* > 0.05

Statistically significant difference: *p* < 0.001

After implementation, statistically significant differences were found among the three groups in all dimensions of self-directed learning readiness and the total score (*p* < 0.001). The video-assisted PBL group achieved the highest mean scores in self-management (38.42 ± 1.50), desire for learning (35.30 ± 1.62), self-control (44.66 ± 1.39), and total self-directed learning readiness (118.38 ± 3.76), followed by the traditional PBL group (37.02 ± 3.91, 34.00 ± 4.00, 42.82 ± 4.73, and 113.84 ± 11.72, respectively). Meanwhile, the control group showed the lowest improvement in all dimensions and the total score (27.92 ± 4.22, 25.02 ± 3.53, 31.92 ± 5.20, and 84.86 ± 10.28, respectively). These findings indicate that both PBL approaches significantly enhanced students’ self-directed learning readiness; however, video-assisted PBL was more effective in improving students’ readiness for self-directed learning compared with traditional PBL and conventional teaching.

Table 9: Post hoc LSD analysis showed no significant differences among groups before implementation (*p* > 0.05). After implementation, significant differences were observed between all groups, with the video-assisted PBL group achieving the highest self-directed learning readiness scores, followed by the traditional PBL group and the control group (*p* < 0.001).

**Table 9:** LSD post hoc multiple comparisons of self-directed learning readiness scores among the video-assisted PBL, traditional PBL, and control groups pre and post implementation (*n* = 150)

Time point	(I) group	(J) group	Mean Difference (I-J)	*P*-value
**before implementation**	Video-assisted PBL group	Traditional PBL group	0.36	0.198
	Video-assisted PBL group	Control group	−0.68	0.160
	Traditional PBL group	Video-assisted PBL group	−0.36	0.198
	Traditional PBL group	control group	−1.04	0.904
	Control group	Video-assisted PBL group	0.68	0.160
	Control group	Traditional PBL group	1.04	0.904
**after implementation**	Video-assisted PBL group	Traditional PBL group	4.54	< 0.001
	Video-assisted PBL group	Control group	33.52	< 0.001
	Traditional PBL group	Video-assisted PBL group	−4.54	< 0.001
	Traditional PBL group	Control group	28.98	< 0.001
	Control group	Video-assisted PBL group	−33.52	< 0.001
	Control group	Traditional PBL group	−28.98	< 0.001

*LSD* Least Significant Difference post hoc test

No statistically significant difference: *p* > 0.05

Statistically significant difference: *p* < 0.001

Table 10: demonstrates the correlation coefficients among students’ total knowledge, academic self-efficacy, and self-directed learning readiness scores before and after implementation in the video-assisted PBL, traditional PBL, and control groups.

**Table 10:** Correlation coefficients among total knowledge, academic self-efficacy, and self-directed learning readiness scores among the study and control groups before and after implementation (*n* = 150)

Time point	Groups	Variables		Total knowledge	Total academic self-efficacy	Total self-directed learning readiness
**Before Implementation**	**Video-assisted PBL group**	**Total knowledge**	r/P-value	1	r = 0.91, p < 0.001	r = 0.84, p < 0.001
		**Total academic self-efficacy**	r/P-value	r = 0.91, p < 0.001	1	r = 0.96, p < 0.001
		**Total self-directed learning readiness**	r/P-value	r = 0.00, p = 0.841	r = 0.96, p < 0.001	1
	**Traditional PBL group**	**Total knowledge**	r/P-value	1	r = 0.87, p < 0.001	r = 0.74, p < 0.001
		**Total academic self-efficacy**	r/P-value	r = 0.87, p < 0.001	1	r = 0.91, p < 0.001
		**Total self-directed learning readiness**	r/P-value	r = 0.74, p < 0.001	r = 0.91, p < 0.001	1
	**Control group**	**Total knowledge**	r/P-value	1	r = 0.92, p < 0.001	r = 0.32, p = 0.022
		**Total academic self-efficacy**	r/P-value	r = 0.92, p < 0.001	1	r = 0.36, p = 0.009
		**Total self-directed learning readiness**	r/P-value	r = 0.32, p = 0.022	r = 0.36, p = 0.009	1
**After Implementation**	**Video-assisted PBL group**	**Total knowledge**	r/P-value	1	r = 0.80, p < 0.001	r = 0.93, p < 0.001
		**Total academic self-efficacy**	r/P-value	r = 0.80, p < 0.001	1	r = 0.94, p < 0.001
		**Total self-directed learning readiness**	r/P-value	r = 0.93, p < 0.001	r = 0.94, p < 0.001	1
	**Traditional PBL group**	**Total knowledge**	r/P-value	1	r = 0.85, p < 0.001	r = 0.89, p < 0.001
		**Total academic self-efficacy**	r/P-value	r = 0.85, p < 0.001	1	r = 0.85, p < 0.001
		**Total self-directed learning readiness**	r/P-value	r = 0.89, p < 0.001	r = 0.85, p < 0.001	1
	**Control group**	**Total knowledge**	r/P-value	1	r = 0.97, p < 0.001	r = 0.98, p < 0.001
		**Total academic self-efficacy**		r = 0.97, p < 0.001	1	r = 0.97, p < 0.001
		**Total self-directed learning readiness**	r/P-value	r = 0.98, p < 0.001	r = 0.97, p < 0.001	1

r = Pearson correlation coefficient

Statistically significant correlation: *p* < 0.05

Highly statistically significant correlation: *p* < 0.001

Before implementation, significant positive correlations were observed between total knowledge and academic self-efficacy scores in the video-assisted PBL, traditional PBL, and control groups (*r* = 0.914, 0.868, and 0.918, respectively; *p* < 0.001). Moreover, significant positive correlations were found between academic self-efficacy and self-directed learning readiness scores in both PBL groups (r = 0.964 and 0.909, respectively; *p* < 0.001), while weaker but statistically significant correlations were observed in the control group (*r* = 0.364, *p* = 0.009).

After implementation, significant positive correlations were detected among all studied variables across the three groups (*p* < 0.001). In the video-assisted PBL group, strong positive correlations were found between knowledge and academic self-efficacy (*r* = 0.798), knowledge and self-directed learning readiness (*r* = 0.929), and academic self-efficacy and self-directed learning readiness (*r* = 0.937). Similar strong positive associations were observed in the traditional PBL group. The control group also demonstrated significant positive correlations among all variables after implementation, with the strongest correlations observed between knowledge and self-directed learning readiness (*r* = 0.978) and between academic self-efficacy and self-directed learning readiness (*r *= 0.974).

These findings indicate that higher levels of knowledge were associated with greater academic self-efficacy and improved self-directed learning readiness among students. The consistent positive correlations after implementation suggest that enhancing students’ knowledge through PBL approaches may contribute to improvements in their confidence and readiness for self-directed learning.

Recent research highlights the pivotal role of problem-based learning (PBL) in nursing education, emphasizing its effectiveness in promoting critical thinking, problem-solving skills, and lifelong learning competencies among students. This educational approach encourages active participation, collaborative learning, and self-directed inquiry by employing authentic clinical scenarios that mirror real-world professional practice. Additionally, incorporating technological tools, particularly video-assisted PBL, enhances the learning environment by making it more dynamic and interactive, thereby improving students’ understanding, engagement, and retention of knowledge [51–56]. Thus, the aim of the present study was to evaluate the effect of traditional versus video-assisted problem-based learning on pediatric nursing students’ academic self-efficacy and self-directed learning readiness.

The current research evaluated the comparative impact of video-integrated versus conventional Problem-Based Learning (PBL) on nursing students' cognitive outcomes, academic self-efficacy, and self-directed learning readiness. Results demonstrated that while both modalities yielded statistically significant gains, the video-assisted approach facilitated more robust and holistic improvements across all evaluated parameters. This suggests that the incorporation of visual technology within PBL frameworks offers a more effective pedagogical strategy for nursing education than traditional methods.

The findings indicated that the majority of participants across both study groups (video-assisted PBL and traditional PBL) and the control group were aged between 20 and 22 years, with closely comparable mean ages. This distribution is consistent with the structure of the Egyptian educational system, where students typically enroll in the third academic year of nursing within this age range. Similar age patterns were reported by Mohamed Ahmed Maiz et al. [57] and Zaki et al. [58].

Regarding gender, females constituted the majority across all groups, reflecting the traditionally higher enrollment of females in nursing education in Egypt. This finding aligns with prior research by Towfik et al. [59] and El-Guindy et al. [60], although it contrasts with Falahan et al. [61], who reported a predominance of male participants.

In terms of residence, most students were from rural areas, which corresponds to the demographic profile of students at Benha University and is supported by earlier studies such as Thabet et al. [62] and Towfik et al. [59], while differing from findings reported by Najjar et al. [63].

Additionally, approximately two-thirds of students held a secondary school certificate prior to university admission, consistent with the standard educational pathway in Egypt, as noted by Zaki et al. [58] and Aly et al. [64]. Finally, none of the participants had received prior training in problem-based learning, which may be attributed to the limited integration of PBL training programs within undergraduate nursing curricula. This finding is consistent with Abdel Moneem et al. [65] and Kansal et al. [66], though it contradicts Najjar et al. [63].

Concerning total nursing students' knowledge level in both study and control groups regarding problem-based learning and selected teaching content, the current study findings represented that, prior to the intervention, most students in the video-assisted PBL, traditional PBL, and control groups demonstrated low levels of knowledge, with no significant differences among the groups. Following the intervention, knowledge scores increased significantly in both the traditional PBL and video-assisted PBL groups compared with the control group.

These results can be explained by the fact that the significant leap in knowledge, particularly in the video-assisted group, can be attributed to the structured and intensive nature of the intervention. First, the preliminary workshop provided a solid foundation, orienting students to PBL principles before the actual application. Second, the adoption of Barrows’ six-step PBL model implemented over two sessions per week allowed for a deep cognitive engagement; the first session focused on brainstorming and identifying gaps, while the second focused on self-study and clinical application, ensuring the transition from theoretical "knowing" to practical "applying."

Furthermore, the repetition of this strategy over five weeks for various pediatric cases created a "cumulative learning effect," where students became more proficient in the problem-solving process with each scenario. The use of AI-designed videos specifically enhanced this process by providing realistic, "ill-structured" clinical problems that stimulated the students' senses and improved their retention of complex pediatric information compared to traditional text-based scenarios.

These results are consistent with the findings of Gandhi and Dass [67], who evaluated the impact of a PBL module on nursing students' knowledge and attitudes. Their baseline assessment revealed that the majority of students possessed only moderately adequate knowledge, with approximately one-quarter showing inadequate levels. However, following the intervention, they reported a statistically significant increase in both knowledge scores and professional attitudes among the participants.

Similarly, Ayomi et al. [68] emphasized that the PBL method is highly effective in increasing students' knowledge compared to traditional methods. Furthermore, these findings align with a growing body of evidence supporting the efficacy of Problem-Based Learning (PBL) over conventional pedagogical methods. Specifically, Amin et al. [69] demonstrated that PBL significantly enhances knowledge acquisition among nursing students compared to traditional lectures. This is further reinforced by a systematic review conducted by Waqar et al. [70], which attributes superior information retention and clinical reasoning skills to the PBL framework. Moreover, the success of integrating structured clinical scenarios such as the pediatric cases utilized in this study is validated by Zhao et al. [71], who reported significant improvements in both quiz performance and basic knowledge following PBL sessions. These consistent findings support the effectiveness of PBL in enhancing learning outcomes in nursing education.

The implementation of Problem-Based Learning (PBL) appears to significantly bolster students’ self-efficacy, a phenomenon largely attributed to its emphasis on active problem-solving and higher-order critical thinking. By shifting the educational focus toward a student-centered, collaborative framework, PBL cultivates the requisite confidence for autonomous knowledge application and academic accountability, as noted by Fitriani et al. [72].

These theoretical underpinnings are empirically supported by the current study’s findings, which indicated that, prior to the intervention, there were no significant differences among the groups in total scores for academic self-efficacy, including its domains of task-difficulty preference, self-confidence, and self-regulatory efficacy. Following the intervention, both the video-assisted and traditional PBL groups demonstrated a highly significant increase in mean scores across all domains. This finding reflects that engaging students in active, problem-centered learning enhances pediatric nursing students’ confidence in their ability to tackle complex tasks. The improvement can be attributed to the collaborative nature of PBL, which allows students to exchange ideas, provides mutual support, and builds confidence through group problem-solving.

In line with the current study, Amin et al. [69] in their quasi-experimental study reported that academic self-efficacy levels were low across all groups at the pre-intervention baseline, with no statistically significant differences observed between the groups. Furthermore, the results of the present study correspond with Abdel Moneem et al. [65], who evaluated the effect of PBL training on newly graduated nurses and determined that, before the PBL training, the participants’ general self-efficacy scores indicated a moderate level of self-efficacy.

Similarly, Lim [73] demonstrated that simulation-linked problem-based learning significantly enhanced nursing students’ learning self-efficacy and critical thinking disposition. Additionally, the current study's finding is supported by Choi et al. [74], who highlighted that PBL significantly enhanced students’ self-efficacy and was positively correlated with improved problem-solving ability, highlighting its effectiveness in fostering confidence and practical skills.

Besides, studies by Yang and Oh [75] and Saepuloh et al. [76] confirm that PBL significantly enhances self-efficacy and critical thinking compared to traditional methods. Specifically, video-assisted PBL fosters confidence by simulating realistic clinical scenarios, effectively bridging the gap between theory and practice.

Problem-based learning (PBL) has been shown to enhance self-directed learning readiness and problem-solving skills compared to lecture-based approaches. It also improves critical thinking and communication, fostering greater student autonomy. Zhao et al. [77] provided meta-analytic evidence supporting the effectiveness of PBL in promoting self-directed learning across diverse educational contexts. This matches with the results of the present study, which illustrated that, prior to the intervention, there were no significant differences among the video-assisted PBL, traditional PBL, and control groups in the mean scores of self-directed learning readiness, including its domains of self-management, desire for learning, and self-control. Following the intervention, both the video-assisted and traditional PBL groups showed highly significant improvements across all domains compared with the control group. Notably, the video-assisted PBL group achieved the highest post-intervention total mean score.

The observed improvement in students’ self-directed learning readiness, particularly in self-management, learning motivation, and self-control, may be attributed to the structured and iterative design of the five-week Problem-Based Learning (PBL) intervention. Grounded in Barrows’ Model, the intervention shifted the locus of control from instructor-led teaching to learner-centered engagement. The transition from problem analysis to independent inquiry required students to develop effective time management and self-regulation skills. Additionally, the use of high-fidelity, AI-generated video scenarios provided an authentic clinical context that facilitated the integration of theoretical knowledge with practice. This approach enhanced students’ intrinsic motivation and maintained engagement across pediatric nursing topics.

Furthermore, the repeated two-session cycle supported metacognitive development by encouraging students to reflect on and refine their problem-solving strategies, thereby strengthening their self-control over learning processes. Finally, the researchers’ role as facilitators fostered a mastery-oriented learning environment, enabling students to transition from passive learners to active, autonomous, and self-directed clinical decision-makers.

These results are consistent with the findings of Chikeme et al. [78], who highlighted that, following the intervention, the PBL group showed a noticeable improvement in self-directed learning readiness, while the traditional teaching group exhibited little change. Additionally, the findings of the current study are in agreement with Xue et al. [79], who demonstrated that problem-based learning significantly enhances nursing students’ self-directed learning ability, with PBL participants showing markedly higher outcomes compared to those receiving traditional instruction. These results are supported by the study performed by Han [80], who showed that mobile-assisted PBL significantly improved university students’ self-directed learning readiness. Similarly, Wong and Kan [81] revealed that the intervention group showed significant and sustained improvements in self-directed learning after structured online small-group work. Moreover, Clausen [82] illustrated that problem-based learning promotes self-directed learning, with students showing increased self-regulation and internal locus of control over time.

The result of the present study revealed that there was a highly statistically significant positive correlation between total knowledge, total academic self-efficacy, and total self-directed learning readiness in all three groups pre- and post-implementation. The observed statistically significant positive correlation between these constructs can be explained by their interdependent nature. Increased knowledge enhances students’ confidence in their academic abilities, thereby strengthening academic self-efficacy. In turn, higher self-efficacy promotes greater motivation and autonomy in learning, which directly supports self-directed learning readiness. The implementation of the PBL program likely contributed to these positive relationships by actively engaging students in realistic clinical scenarios that required critical thinking, problem-solving, and independent decision-making. PBL encourages students to take responsibility for their own learning, seek relevant information, and apply knowledge in context, which simultaneously reinforces understanding, boosts confidence in academic capabilities, and fosters readiness for self-directed learning. Thus, PBL serves as an effective pedagogical approach that enhances knowledge acquisition while simultaneously promoting self-efficacy and autonomous learning behaviors.

Ma et al. [83] reported that academic self-efficacy positively and significantly influences nursing students’ self-directed learning ability, with learning engagement serving as a mediating mechanism. The current findings are consistent with this observation. Therefore, it is essential for nursing educators to implement strategies that enhance students’ academic self-efficacy and actively promote learning engagement to strengthen their self-directed learning skills. Similarly, Meng et al. [84] reported that academic self-efficacy, positive learning attitudes, and professional interest were positively correlated with nursing students’ self-directed learning readiness. Also, these results correspond with Harjanto et al. [85], who found a significant positive correlation between SDLR and overall online self-efficacy, indicating that higher readiness is associated with greater confidence in completing online learning tasks. Additionally, Mi and Nwe [85] showed a strong positive correlation between self-directed learning and academic self-efficacy among education college students. Furthermore, the finding of the present study is compatible with Yang and Oh [75], who demonstrated a positive relationship among the variables, showing that higher learning motivation and engaging multimedia video-assisted PBL scenarios are associated with increased academic self-efficacy and self-directed learning.

### Innovation

The findings of this study offer substantive evidence for advancing pediatric nursing education by integrating innovative, technology-driven pedagogical strategies. Beyond confirming the efficacy of Problem-Based Learning (PBL), these results highlight the transformative potential of AI-generated, video-assisted PBL in elevating students’ cognitive achievement, academic self-efficacy, and self-directed learning readiness.

The shift from traditional, teacher-centered instruction toward this dynamic, student-centered environment fosters a more immersive learning experience. By presenting realistic, high-fidelity pediatric clinical scenarios, the video-assisted approach stimulates higher-order cognitive functions, particularly clinical reasoning and critical thinking, which are essential for navigating complex pediatric cases.

Furthermore, the integration of AI-video technology bridges the persistent gap between theoretical knowledge and clinical reality. The visualization of intricate pediatric conditions through digital avatars enhances information retention and provides a safe, controlled environment for students to develop decision-making and problem-solving skills. Such a model not only boosts clinical preparedness but also nurtures professional competencies, including self-regulation and autonomous learning traits that are indispensable for modern healthcare providers.

From a clinical perspective, adopting video-assisted PBL prepares pediatric nursing students to manage real-world healthcare challenges with greater confidence and precision. It equips them with the necessary tools to deliver safe, high-quality, and patient-centered care, ultimately contributing to improved pediatric health outcomes.

Consequently, this study advocates for a strategic curriculum redesign that incorporates technology-enhanced PBL. Such an innovative educational model aligns with the evolving demands of contemporary healthcare systems and ensures that future pediatric nurses are both competent and resilient in their professional practice.

### Limitations

Several limitations should be considered when interpreting the findings of this study. First, the study was conducted at a single academic institution (Faculty of Nursing, Benha University), which may limit the generalizability of the findings to nursing students in different educational and cultural contexts. Therefore, future multicenter studies are recommended to confirm the applicability of the findings across diverse settings.

Second, the intervention was implemented within the constraints of the regular academic schedule, which may have limited the amount of time available for extended discussion, reflection, and individualized feedback during some learning sessions.

Third, although students were assigned to separate groups, the possibility of informal communication and information exchange between participants in the intervention and control groups could not be entirely eliminated. Such contamination is a common challenge in educational research conducted within the same institution.

Fourth, academic self-efficacy and self-directed learning readiness were assessed using self-reported instruments, which may be subject to social desirability and response biases.

Fifth, the study assessed outcomes immediately following the intervention. Therefore, the long-term retention of knowledge and the sustainability of improvements in academic self-efficacy and self-directed learning readiness were not evaluated.

Finally, although the final sample size was reduced due to participant non-consent and withdrawals, post hoc power analysis demonstrated that the analyzed sample retained adequate statistical power to detect the observed effects. In addition, the development of high-quality AI-generated educational videos required considerable technical expertise and preparation time, which may present practical challenges for large-scale implementation in some educational settings.

## Conclusion

The present study concludes that Problem-Based Learning (PBL), particularly when augmented with AI-generated clinical videos, significantly outperforms traditional teaching methods in enhancing pediatric nursing students' knowledge, academic self-efficacy, and self-directed learning readiness. The strong positive correlations observed between these domains suggest that technology-enhanced PBL creates a synergistic effect, where improved self-confidence directly bolsters autonomous learning and academic achievement [86].

These findings underscore the potential of integrating immersive visual tools into nursing curricula to bridge the gap between theoretical instruction and clinical reality. As nursing education evolves toward competency-based models, adopting scalable, video-assisted PBL approaches offers a sustainable pathway for developing highly skilled, autonomous, and resilient healthcare professionals. Future research should focus on longitudinal assessments across multiple institutions to evaluate the long-term sustainability of these educational gains in real-world clinical practice.

## Supplementary Information

Supplementary Material 1.

## Data Availability

The datasets used and/or analyzed during the current study are available from the corresponding author on reasonable request.
