# Supplementary material for: Effect of traditional versus video-assisted problem-based learning on pediatric nursing students’ academic self-efficacy and self-directed learning: implications for pediatric patients care
Source: BMC Med Educ. 2026 Aug 11;26:1303. doi: 10.1186/s12909-026-10037-9 (PMC13465185; doi:10.1186/s12909-026-10037-9)
Supplement: Supplementary file 1 [file 12909_2026_10037_MOESM1_ESM.docx]

**Tool (I): A structured interviewing questionnaire:**

**Dear nursing student**

This questionnaire sheet aim to assess nursing students’ knowledge about problem based learning; you are kindly requested to choose the correct answer. You are crucial for finalization of this work knowing that this information is gathered for scientific research only.

**Part I: Personal characteristics data:**

**Name: (optional)………………………….** **Code number**

1- **Age**
1) Less than 20 years	(	)
2) From 20 < 22 years	(	)
3) From 22 < 24 years	(	)
4) More than 24 years	(	)
2- **Gender :**
1) Male	(	)
2) Female	(	)
3- **Place of residence:**
1) Urban	(	)
2) Rural	(	)
4- **Qualification before attachment to the faculty:**
1) Associate degree of nursing	(	)
2) Secondary school	(	)
5- **Attending previous training program about problem based learning:**
1) Yes	(	)
2) No	(	)

**Part (II): Students' knowledge assessment questionnaire:**

**A: Knowledge of the pediatric nursing students about the problem based learning**

**Dear student**

In this questionnaire, it consists of several elements of problem based learning, there is a list of questions to assess your knowledge about problem based learning and please answers the following questions carefully and read every statement.

**Section one: Multiple choices**

**Select the best answer:**

1. **What is the problem-based learning?**

a) It is an old method of teaching.
b) It is teacher-center learning.
c) It is lecture that emphasis on giving information.
d) It is a new method of teaching used as a tool to achieve both the required knowledge base and skills.
e) Don't know
   2. **What is the core element of the problem-based learning design?**
a) The instructor's lecture
b) The textbook content
c) The problem itself
d) The final exam
e) Don't know
   3. **What are characteristics of the problem-based learning?**
a) It is teacher centered
b) It occurs in large student groups
c) Teachers serve as source of new information
d) Teachers serve as facilitators
e) Don't know
   4. **What is the goal of the problem-based learning?**
a) Develop self-directed, lifelong learning skills.
b) presentation of large amount of information
c) Prepare student to pass final exam effectively
d) presentation of educational content to large student groups
e) Don't know
   5. **What is the appropriate number of students for the problem-based learning session?**
a) 8-10
b) 12-15
c) 15-20
d) 20-25
e) Don't know
   6. **What is the teacher role in the problem-based learning?**
a) Teaching
b) Learning
c) Facilitator
d) Leader
e) Don't know
   7. **Who is the person responsible for taking notes, summarizing team discussions?**
a) Facilitator
b) Recorder
c) Reporter
d) Timekeeper
e) Don't know

8. **Who is the person responsible for spoken to the class or instructor, summarizing the group's activities and/or conclusions?**

a) Facilitator
b) Recorder
c) Reporter
d) Timekeeper
e) Don't know
   9. **Who is the person responsible for making sure that group focuses on most important issues?**
a) Explorer
b) Prioritizer
c) Reporter
d) Timekeeper
e) Don't know
   10. **What is the role of the innovator during the problem-based learning session**?
a) Encourages imagination and contributes new and alternative perspectives and ideas.
b) Receive information from teacher
c) understanding the content being presented by teacher
d) prepare and review the educational material before the class
e) Don't know
   11. **What is the first step at the problem-based learning?**
a) Developing a PBL problem scenario
b) Identifying key information
c) Presentation of the problem/case
d) Development of objectives
e) Don't know
   12. **What is the second step of problem-based learning?**
a) Developing a PBL problem scenario
b) Identifying key information
c) Development of objectives
d) Presentation of the problem/case
e) Don't know
   13. **What is the next step after PBL scenario presentation?**
a) Go and learn
b) Reporting back
c) Identifying key information
d) Presentation
e) Don't know
   14. **In which step team members present their searching regard PBL scenario?**
a) Go and learn
b) Reporting back
c) Identifying key information
d) Presentation
e) Don't know
   15. **In which step team members go away to research about problem?**
a) Reporting back
b) Identifying key information
c) Presentation
d) Go and learn
e) Don't know
   16. **What is the last step in the problem-based learning?**
a) Reporting back
b) Identifying key information
c) Presentation
d) Action/integrating new knowledge
e) Don't know
   17. **What is the appropriate time to be available between tutorials?**
a) 2-3 day
b) 5-7 days
c) 7-9 days
d) More than 10 days
e) Don't know
   18. **What are advantages of the problem-based learning?**
a) Evaluation is quite difficult
b) Resource-expensive
c) developing lifelong learning skills
d) very difficult and expensive to use when class size is large
e) Don't know
   19. **What are disadvantages of the problem-based learning?**
a) It helps in developing lifelong learning skills
b) Increases ability to apply knowledge in clinical situations
c) Measurement of learning outcomes is difficult
d) Increased motivation for learning
e) Don't know
   20. **What are difficulties which may be in PBL in group?**
a) The dominating group member
b) Cooperation between team member
c) Proper time management by team members
d) Good preparation for the tutorial
e) Don't know

**B: Knowledge of the pediatric nursing students after application of the problem based learning regarding the selected teaching content of pediatric nursing course**

**A**) Knowledge of the pediatric nursing students regarding Diabetes

1. **Diabetes is defined best as...**
a. A metabolic disease characterized by low blood sugar
b. A metabolic disease characterized by high blood sugar
c. A family of blood infections
d. None of the above
e. Don't know
2. **Insulin is a natural hormone secreted by which organ or gland?**
a. The kidneys
b. The liver
c. The pancreas
d. The spleen
e. Don't know
3. **What is the function of insulin?**
a. Lets blood sugar leave the liver
b. Helps cells take in blood sugar
c. Helps cells keep out blood sugar
d. Lets body release adrenaline
e. Don't know
4. **Which type of diabetes called Insulin-dependent diabetes?**
a. [Type 1 diabetes](https://en.wikipedia.org/wiki/Type_1_diabetes)
b. [Type 2 diabetes](https://en.wikipedia.org/wiki/Type_2_diabetes)
c. Gestational diabetes
d. None of the above
e. Don't know
5. **The risk factors for type 1 diabetes include:**
a. Diet
b. age
c. Autoimmune
d. Environmental
e. Don't know.
6. **Which of the following best describes Type 2 diabetes?**
a. pancreas produce enough insulin
b. juvenile diabetes
c. loss of beta cells is caused by an [autoimmune](https://en.wikipedia.org/wiki/Autoimmunity) response
d. a condition in which cells fail to respond to insulin properly
e. Don't know
7. **The risk factors for type 2 diabetes mellitus include:**
   a. autoimmune destruction pancreas
   b. being overweight
   c. high intake of vegetables
   d. Advanced age
   e. Don't know
8. **Which statement best describes the differences between the characteristics of type 1 and type 2 diabetes:**
   a. persons with type 2 diabetes usually require lower doses of insulin than person with type 1 diabetes because they have a milder form of diabetes
   b. persons with type 1 diabetes rapidly develop chronic complications
   c. autoimmune factors are involved in the pathogenesis of type 1 but not type 2 diabetes
   d. persons with type 1 diabetes can increase endogenous insulin production by taking oral hypoglycemic agents

e.	Don't know

9. **Which of the following confirmed values meet the diagnostic threshold for diabetes?**
   a. Fasting blood glucose = 140 mg/dl
   b. random glucose > 160 mg/dl
   c. 2 hour post prandial glucose ≥ to 126 mg/dl
   d. fasting blood glucose ≥ 126 mg/dl
   e. Don't know
10. **The test for checking mean plasma glucose concentration over the previous 8-10 weeks is:**
    a. Hemoglobin A1c
    b. Oral glucose tolerance test (OGTT)
    c. Fructosamine test
    d. Fasting plasma glucose concentration
    e. Don't know
11. **Blood sugar is well controlled when Hemoglobin A1C is:**
a. Below 7%
b. Between 12%-15%
c. Less than 180 mg/dL
d. Between 90 and 130 mg/dL
e. Don't know
12. **Among female children and adolescents, the first sign of type 1 diabetes may be:**
a. Rapid weight gain
b. Constipation
c. Genital candidiasis
d. Insomnia
e. Don't know
13. **Untreated hyperglycemia may lead to all of the following complications except:**
a. [Diabetic nephropathy](https://en.wikipedia.org/wiki/Diabetic_nephropathy)
b. Vitiligo
c. Diabetic ketoacidosis
d. Dental decay
e. Don't know
14. **According to trials on diabetes prevention, high-risk individuals can reduce their risk to develop diabetes by doing the following:**
    a. Eating a very low carbohydrate diet
    b. Consuming a diet high in monounsaturated fats
    c. Losing 5-7% of body weight through a hypocaloric low fat diet and 30 minutes of daily activity
    d. Initiating metformin 850 mg BID and practicing daily vigorous exercise
    e. Don't know
15. **Which of the following measures don't help to prevent diabetes complications?**
a. Controlling blood glucose
b. Controlling blood pressure and blood lipids
c. Eliminating all carbohydrates from the diet
d. Prompt detection of diabetic eye and kidney disease
e. Don't know
16. **Which of the following regimens offer the best blood glucose control for persons with type 1 diabetes?**
a. A single anti-diabetes drugs
b. Once daily insulin injections
c. A combination of oral anti-diabetic medications
d. Three or four injections per day of different types of insulin.
e. Don't know
17. **The benefits of using an insulin pump include:**
a. Changing infusion sets more difficult than single injections
b. They simplify management of blood sugar and often improve A1C
c. They enable exercise without compensatory carbohydrate consumption
d. They help to weight gain
e. Don't know
18. **Which action should the nurse take after a patient treated with intramuscular glucagon for hypoglycemia regains consciousness?**
a. Assess the patient for symptoms of hyperglycemia
b. Give the patient a snack of peanut butter and crackers.
c. Have the patient drink a glass of orange juice or nonfat milk.
d. Administer a continuous infusion of 5% dextrose for 24 hours.
e. Don't know
19. **A pediatric patient who has type 1 diabetes plans to swim laps for an hour daily at 1:00 PM. The clinic nurse will plan to teach the patient to**
    a. Check glucose level before, during, and after swimming.
    b. Delay eating the noon meal until after the swimming class.
    c. Increase the morning dose of neutral protamine Hagedorn (NPH) insulin.
    d. time the morning insulin injection so that the peak occurs while swimming
    e. Don't know.
20. **The nurse identifies a need for additional teaching when the patient who is self-monitoring blood glucose**
a. Washes the puncture site using warm water and soap.
b. Chooses a puncture site in the center of the finger pad.
c. Hangs the arm down for a minute before puncturing the site.
d. says the result of 120 mg indicates good blood sugar control
e. Don't know

**B**) Knowledge of the pediatric nursing students regarding Asthma

1. **What is an asthma attack?**
a. When the lungs fill with water
b. When airways tighten and the lungs don't get enough air
c. When the heart beats too fast
d. When the heart and lungs are working too hard
e. Don't know
2. **Which of the following is NOT a characteristic of asthma?**
a. Increase in IgG immunoglobulins
b. Airway hyper responsiveness
c. Infiltration of eosinophils into the airways
d. Increased mucus production
e. Don't know
3. **What is a common symptom of asthma?**
a. Wheezing
b. Whistling
c. Full breaths
d. Snoring
e. Don't know
4. **The immediate response of asthma involves**
a. mast cell degranulation
b. binding of antigen to IgE on macrophages
c. release of cytokines such as IL-13
d. Activation of cholinergic nerves.
e. Don't know
5. **What are symptoms of asthma?**
a. Tightness in the chest
b. Wheezing
c. Sneezing
d. A and B
e. Don't know
6. **Which of these can trigger an asthma attack?**
   a. Exercise
   b. Aspirin
   c. Laughing
   d. All of the above
   e. Don't know
7. **Which of these allergens are most likely to bring on an asthma attack?**
   a. Ragweed
   b. Cockroach dander
   c. Dust mites
   d. A, B and C
   e. Don't know
8. **Which of these foods should children with asthma stay away from?**
   a. Dairy products
   b. All nuts
   c. Foods with preservatives
   d. None of the above
   e. Don't know
9. **Which sports are best for children with asthma?**
   a. Baseball
   b. Swimming
   c. Sprinting
   d. All of the above
   e. Don't know
10. **What can you do to reduce exposure to outdoor asthma triggers?**
    a. Exercise outdoors on high ozone days
    b. Schedule camping trips in May, June, or August through October
    c. Pursue outdoor sports in cold weather
    d. None of the above
    e. Don't know
11. **Which of these is a good idea when a child with asthma is outside in cold weather?**
    a. Wear a scarf to cover the nose and mouth
    b. Wear extra clothing to stay warm
    c. Check pollution levels in the area
    d. Keep hands away from the nose and mouth
    e. Don't know
12. **What should you do if pets trigger your child's asthma?**
    a. Bathe the pet weekly
    b. Keep the pets out of your child's bedroom
    c. Have your child wash their hands after touching pets
    d. All of the above
    e. Don't know
13. **What can nurse educate parents to reduce asthma triggers in your home?**
a. Clean up mold with soap and water
b. Don’t smoke in the home
c. Vacuum carpets, rugs and furniture often
d. All of the above
e. Don't know
14. **Which of the following is not a goal of asthma treatment?**
a. To cure asthma
b. To control airway swelling
c. To learn about your asthma triggers so you can avoid them
d. To control secondary infection
e. Don't know
15. **What are the main types of medicine for asthma?**
a. Medicines that work fast (quick-relief drugs) to ease asthma symptoms
b. Medicines that work over time (control drugs) to prevent asthma attacks
c. Both A and B
d. Analgesics
e. Don't know
16. **What the device used with an inhaler to help get more medicine into the airways?**
a. Spacer
b. Cap
c. Mouthpiece
d. Canister
e. Don't know
17. **Which action should be included in asthma action plan?**
    a. A plan for taking asthma medicines when child condition is stable
    b. A list of asthma triggers and how to avoid them
    c. How to tell when your asthma gets worse, and when to call doctor or nurse
    d. All of the above
    e. Don't know
18. **Which of these asthma symptoms is considered an emergency?**
    a. Bluish lips and face
    b. Extreme difficulty breathing
    c. Severe anxiety caused by shortness of breath
    d. All of the above
    e. Don't know
19. **Which of the following instructions can prevent asthma symptoms after exercise?**
a. avoid [exercise](https://www.webmd.com/fitness-exercise/ss/slideshow-7-most-effective-exercises) because of exercise-induced asthma
b. Inhalation medications taken prior to exercise
c. outside exercise available even high [pollen](https://symptoms.webmd.com/seasonal-allergy-map-tool/pollen_allergies_overview) days
d. outside exercise available at cold days
e. Don't know
20. **The 10 year old child is being treated for asthma. Which important health education should be given to the child?**
    a. Exercising in a cold or dry air.
    b. Covering the child with a wool blanket.
    c. Avoiding crowding places.
    d. Avoiding triggers as tobacco smoke.
    e. Don't know

**C) Knowledge of the pediatric nursing students regarding Rheumatic Fever**

1. **What is rheumatic fever?**
a. Strep throat infection
b. Inflammatory disease
c. Rheumatic infection
d. Rheumatoid arthritis
e. Don't know
2. **Rheumatic fever is caused by which of the following bacteria?**
   a. Pseudomonas
   b. Staphylococcus
   c. Streptococcus
   d. Shingella
   e. Don't know
3. **Rheumatic fever most commonly affects children/or adolescents aged ……. years.**
a. 1 to 4
b. 5 to 15
c. 16 to 18
d. 18 to 20
e. Don't know
4. **In children younger than 10 years of age, which gender is more often affected by chorea associated with rheumatic fever?**
   a. Boys are affected more than girls
   b. Girls are affected more than boys
   c. Boys and girls are equally affected
   d. Younger girls more affected
   e. Don't know
5. **Which of the following factors in the patient’s history and physical examination is least relevant to the diagnosis of rheumatic fever?**
   a. sore throat from  3 weeks previous
   b. living in a crowded or poor environment
   c. the mother’s history of type-2 diabetes
   d. a possible genetic factor from the father who’s history is unknown
   e. Don't know
6. **To diagnose rheumatic fever, evidence of preceding group A streptococcal infection may include which of the following?**
a. Positive throat culture or rapid strep test
b. Blood culture
c. Urine culture
d. A and B
e. Don't know
7. **Which of the following is a minor sign of rheumatic fever?**
   a. Arthralgia.
   b. Carditis.
   c. Polyarthritis.
   d. Chorea.
   e. Don't know
8. **Which of the following is usually the first symptom of rheumatic fever?**
a. Chest pain or palpatations
b. Jerky, uncontrollable movements
c. Joint pain and fever
d. Rash
e. Don't know
9. **Which is NOT a symptom of rheumatic fever?**
a. Painless rash, joint pain
b. Fever, fatigue
c. Joint pain, Sydenham chorea
d. Swollen tonsils
e. Don't know
10. **While most people with rheumatic fever recover, which part of the body may be permanently damaged in a small percentage of people?**
a. Digestive tract
b. Ears
c. Heart
d. Lungs
e. Don't know
11. **According to the Jones Criteria, which of the following sets of symptoms would indicate a positive diagnosis for Rheumatic fever? Select all that apply.**
    a. Carditis, fever, and an elevated WBC count
    b. Positive strep throat culture, arthritis, chorea
    c. Recent scarlet fever, carditis, fever, arthralgia
    d. Elevated C-reactive protein, carditis, fever
    e. Don't know
12. **Which of the following symptom is NOT included in the major criteria?**
a. Erythema marginatum
b. Subcutaneous nodules
c. Carditis
d. Elevated acute phase reactants
e. Don't know
13. **What can prevent rheumatic fever?**
a. Childhood vaccinations
b. Developed immunity
c. Treating strep throat
d. Avoiding ill people
e. Don't know
14. **Each of the following drugs is used to treat rheumatic fever in children EXCEPT:**
a. Acetaminophen
b. Antibiotics
c. Anti-inflammatory drugs
d. Corticosteroids
e. Don't know
15. **all the following are prophylactic treatment of rheumatic fever except:**
a. Treatment of any streptococcal infection
b. Tonsillectomy for chronically infected tonsils
c. Long acting penicillin
d. Salicylate
e. Don't know

**D) Knowledge of the pediatric nursing students regarding Thalassemia**

1. **Which of the following statements is true about thalassemia?**
a) Thalassemia affects red blood cells, turning them from round flexible discs into stiff form
b) Thalassemia is caused by the mutations in DNA of cells that form the hemoglobin.
c) Thalassemia is a rare disorder in which the blood doesn't clot in the typical way.
d) All the above.
e) Don't know.
2. **How many amino acid chains are found in hemoglobin?**
a) One
b) Two
c) Three
d) Four
e) Don't know
3. **Which blood tests can tell if a person is a thalassemia carrier?**
a) Complete Blood Count (CBC)
b) Prenatal Testing
c) Reticulocyte Counts
d) All the above
e) Don't know
4. **Which of the following factors leads to an increase in the risk of thalassemia disease**
a) infection
b) Family history of thalassemia
c) bleeding
d) Both A and B
5. **The nursing student is presenting a clinical conference and discusses the cause ofβ-thalassemia. The nursing student informs the group that a child at greatest risk of developing this disorder is which of these?**
a) A child of Mexican descent
b) A child of Mediterranean descent
c) A child whose intake of iron is extremely poor
d) A breast-fed child of a mother with chronic anemia
e) Don't know
6. **Which of the following types of thalassemia disease is Cooley Anaemia?**
a) Thalassemia minor
b) Alpha – Thalassemia
c) Beta – Thalassemia
d) None of the above
e) Don't know.
7. **Which of the following statements is correct about beta – thalassemia is correct?**
a) affects one or both of the beta- globin genes
b) affects the alpha-globin gene(s)
c) More frequent in China, India
d) All the above
e) Don't know
8. **Which of the following is not a symptom of Thalassemia Disease?**
a) Abdominal cramps
b) Weakness and slow growth
c) Dark urine
d) Facial bone deformities
e) Don't know.
9. **Which of the following types of thalassemia is characterized by severe symptoms of anemia such as fatigue, weakness, jaundice and gallstones, and sometimes an enlarged spleen?**
a) Alpha-thalessemia major
b) Alpha-thalessemia minor
c) Beta-thalessemia major
d) Beta-thalessemia minor
e) Don't know
10. **What are the possible complications that can occur in moderate thalassemia?**
a) Iron Overload
b) Infection
c) bleeding
d) Both A & B
e) Don't know
11. **What complications can occur as a result of severe thalassemia?**
a) Deformities in Bone
b) Enlarged Spleen
c) Problems of Heart
d) All the above
e) Don't know
12. **Which of the following is NOT used as a treatment for thalassemia?**
a) Blood transfusions
b) Chemotherapy
c) Iron chelation therapy
d) Removal of the spleen
e) Don't know
13. **When teaching the parents of a child newly diagnosed with beta-thalassemia, which statement made by the parents indicates a need for additional teaching?**
a) “We will give the child daily iron supplements.”
b) “The child will need to have blood transfusions.”
c) “If we want more children, we should have genetic counseling.”
d) “Swimming is a good activity for the child.”
e) Don't know
14. **A nurse is doing discharge education with a parent who has a child with beta-thalassemia (Cooley anemia). The nurse informs the parent that the child is at risk for which of the following conditions?**
a) Hypertrophy of the thyroid.
b) Polycythemia vera.
c) Thrombocytopenia.
d) Chronic hypoxia and iron overload.
e) Don't know
15. **A child with thalassemia was given desferoxamine (Desferal); which of the following should alert the nurse to notify the physician**
a) Decreased hearing
b) Hypertension
c) Red urine
d) Vomiting
e) Don't know

**D) Knowledge of the pediatric nursing students regarding Kwashiorkor**

1. **Kwashiorkor is the disease caused due to the deficiency of-**
a) Vitamins
b) Carbohydrates
c) Minerals
d) Proteins
e) Don't know
2. **A parent asks the nurse to tell her what kwashiorkor is. Which of the following would be the best response of the nurse?**
a) Severe form of [malnutrition](https://my.clevelandclinic.org/health/diseases/22987-malnutrition) specifically, protein-energy under nutrition. It results from an overall lack of calories
b) softening and weakening of bones in children
c) Nutritional disorder occurs as results of a deficiency of protein with an adequate supply of calories.
d) chronic complex disease defined by excessive fat deposits that can impair health
e) Don't know
3. **Which of the following best describes edema of kwashiorkor?**
   a) Starts as puffiness around the eyes, upper limbs, and then becomes generalized.
   b) Caused by hyo-proteinemia.
   c) Is non-pitting edema.
   d) Is usually associated with ascites
   e) Don't know
4. **Nurse explains to parent predisposing factors of kwashiorkor which statement consider correct?**
a) Inadequate [breastfeeding](https://my.clevelandclinic.org/health/articles/5182-breastfeeding) will result in kwashiorkor
b) Excessive use of protein post sudden weaning
c) Depending on diet filled with fats and protein
d) Unbalanced diet with minimal protein and high carbohydrate intake will result in kwashiorkor
e) Don't know
5. **All of the following can be observed in kwashiorkor except?**
a) Generalized edema
b) Failure to gain weight and grow
c) Monkey face appearance
d) Light in color hair which easily packable
e) Don't know
6. **Which of the following symptoms is characteristic of kwashiorkor?**
   a) Decreases in intelligence and learning ability
   b) Energy deficiency
   c) Muscle wasting
   d) Swollen belly
   e) Don't know
7. **Which of the following consider complications of kwashiorkor?**
a) Dehydration
b) Bone deformity
c) Diabetes
d) liver disease
e) Don't know
8. **Which dietary modification is recommended for pediatric patient with kwashiorkor?**
a) High-carbohydrate diet
b) Low-protein diet
c) Calculated protein-carbohydrate diet
d) Low-glycemic index diet
e) Don't know
9. **Treatment protocols for protein-energy malnutrition begin with**
a) Immunization to enhance ability to fight infection.
b) Weight bearing exercise.
c) Replenishment of fluids followed by gradual increase in high quality protein and/or calories in the diet.
d) Supplementation of the diet with a high protein/high calorie drink.
e) Don't know
10. **Infant is being treated for kwashiorkor, which is the important health education should be given to the mother?**
    a) breastfeeding
    b) Nutrition.
    c) Hygiene.
    d) All of the above
    e) Don't know.
